# Extensive Use of 3D Nonfluoroscopic Mapping Systems for Reducing Radiation Exposure during Catheter Ablation Procedures: An Analysis of 10 Years of Activity

**DOI:** 10.1155/2019/4217076

**Published:** 2019-03-10

**Authors:** Massimiliano Marini, Marta Martin, Daniele Ravanelli, Maurizio Del Greco, Silvia Quintarelli, Fabrizio Guarracini, Alessio Coser, Aldo Valentini, Roberto Bonmassari

**Affiliations:** ^1^Department of Cardiology, S. Chiara Hospital, Trento, Italy; ^2^Department of Physics, S. Chiara Hospital, Trento, Italy; ^3^Department of Cardiology, S. Maria del Carmine, Rovereto, Italy

## Abstract

**Purpose:**

3D nonfluoroscopic mapping systems (NMSs) are generally used in the catheter ablation (CA) of complex ventricular and atrial arrhythmias. The aim of this study was to evaluate the efficacy, safety, and long-term effect of the extended, routine use of NMSs for CA.

**Methods:**

Our study involved 1028 patients who underwent CA procedures from 2007 to 2016. Initially, CA procedures were performed mainly with the aid of fluoroscopy. From October 2008, NMSs were used for all procedures.

**Results:**

The median fluoroscopy time of the overall CA procedures fell by 71%: from 29.2 min in 2007 to 8.4 min in 2016. Over the same period, total X-ray exposure decreased by 65%: from 58.18 Gy⁎cm^2^ to 20.19 Gy⁎cm^2^. This reduction was achieved without prolonging the total procedure time. In AF CA procedures, the median fluoroscopy time fell by 85%, with an 86% reduction in total X-ray exposure. In SVT CA procedures, the median fluoroscopy time fell by 93%, with a 92% reduction in total X-ray exposure. At the end of the follow-up period, the estimated probability of disease-free survival was 67.7% at 12 months for AF CA procedures and 97.2% at 3 months for SVT CA, without any statistically significant difference between years.

**Conclusions:**

Our study shows the feasibility of using NMSs as the main imaging modality to guide CA. The extended, routine use of NMSs dramatically reduces radiation exposure, with only slight fluctuations due to the process of acquiring experience on the part of untrained operators, without affecting disease-free survival.

## 1. Introduction

Radiofrequency catheter ablation for the treatment of cardiac arrhythmias [1–3] requires extended X-ray exposure [4], and the relationship between X-ray exposure time and an increased risk of developing cancer has been well established [5–7]. The use of 3D nonfluoroscopic mapping systems (NMSs) for catheter ablation (CA) of many arrhythmias has allowed understanding and ablating complex arrhythmias [8–11] and allowed reducing radiation exposure [12–16]. However, complete elimination or the “near-zero” use of X-ray fluoroscopy during electrophysiological procedures can be achieved only after adequate experience and training [17–20]. We hypothesized that the extensive use of NMSs during CA could yield an overall greater reduction in radiation exposure than its use only in selected cases. 

In a previous work [21] we described our experience of extensive NMSs use in our EP Lab for all CA procedures from 2007 to 2011. In this paper, we extended our observation to the period ranging from 2007 to 2016. We focused on the efficacy, safety, and long-term effect of the extended use of NMSs for CA. We also reported an additional insight into the influence of this new technology on procedures always performed with the aid of NMSs since their introduction into the EP Lab, such as AF CA procedures, and procedures rarely performed with NMSs, such as supraventricular tachycardia (SVT) CA procedures.

## 2. Methods

### 2.1. Study Population

This nonrandomized study involved 1028 consecutive patients (pts) (1145 procedures) who underwent CA procedures from January 2007 to December 2016. Overall, 350,000 residents refer to the EP Lab of our hospital and the total number of CA procedures per year is approximately 140. We report the data on all CA procedures for each year; in 2007 and 2008, some of the procedures were performed by using only fluoroscopy, whereas in subsequent years (from 2009 to 2016) all the procedures were performed by also using a nonfluoroscopic mapping system (NMS). We would like to emphasize that the data for each year were compared with those for each other year and, in particular, with the data from 2007, when the CA procedures were mainly performed with fluoroscopy. We carried out a detailed subanalysis only for AF CA and SVT CA. 

### 2.2. Electrophysiological Procedures: Evolution and Philosophy of Our Lab

In 2001, we started using a single NMS (CARTO, Biosense-Webster, Diamond Bar, CA, USA, Unix Version) in CA procedures for the treatment of complex arrhythmias (atrial fibrillation, atypical atrial flutter, atrial tachycardia, and ventricular tachycardia). In 2007, the new CARTO XP Version allowed us to integrate CT/MRI images of the cardiac chambers with the electroanatomic maps. As a result, images of the left atrium (LA) were integrated during AF ablation or atypical atrial flutter ablation procedures. The following year (2008), we started to use a second NMS (EnSite NavX™ St. Jude Medical, St Paul, MN, USA) for SVT CA (AVNRT, AVRT, atrial tachycardia, atrial flutter). Since October 2008, in agreement with our administration, all CA procedures, with the exception of His-bundle ablation procedures, have been performed by using one or the other of these two NMSs. It should also be specified that, in our EP Lab, all AF CA procedures have always been performed by using the CARTO system, and almost all SVT CA procedures have been performed by using the EnSite system. This use of a specific mapping system for a specific type of ablation procedure was for no other reason than our prior experience and confidence with the specific NMS. New updated hardware and software versions of these NMSs have been used since 2010 (CARTO3 and EnSite Velocity) and since 2015 (EnSite Precision). Fluoroscopy was performed by means of a Toshiba Radiographic/Fluoroscopic Unit (Infinix-I Series CAS, Toshiba Medical System Corporation, Japan). We utilized the same number of catheters during the procedures, regardless of whether fluoroscopy or an NMS was used. Four diagnostic catheters (3 quadripolar and 1 decapolar) and the ablation catheter were used for SVT CA procedures (right jugular vein and right femoral vein as access) and three diagnostic catheters (1 duo-decapolar, 1 quadripolar, and 1 circular mapping catheter) and the ablation catheter were used for AF CA procedures (right jugular vein and right femoral vein as access). From 2007 to 2012, all CA procedures were performed by an experienced operator and by a second operator who was finishing his period of training during the first 3 years of our study. Another two operators started their training in 2013 and 2016, respectively; they were first involved in SVT CA procedures and subsequently in more complex procedures, such as AF CA. During this last period an experienced operator left the lab. We consider an operator who has achieved the minimum procedural volume proposed by ACC/AHA/HRS 2015 Training Statement experienced [22], i.e., 160 catheter ablation procedures.

### 2.3. Procedure and Fluoroscopy Data

In this study, we compared fluoroscopy time, total X-ray exposure, and the duration of each CA procedure. Fluoroscopy time (FT) was defined as the cumulative duration of fluoroscopy during the entire procedure, whereas the patient radiation dose was assessed as the recorded dose-area product (DAP). Procedure time (PT) was measured as the interval from the initial recording of intracardiac signals to the final ECG recording before the end of the procedure.

### 2.4. Procedural Success, Complications and Follow-Up

The definition of procedural success was based on a specific type of tachycardia, as is commonly the international criterion [1, 2]. All patients underwent postprocedural echocardiography to exclude pericardial effusion or other acute complications. We also recorded any other complication that might have occurred during the procedure or during the same hospital stay. We conducted a follow-up outpatient visit at 3 months for SVT CA procedures and at 12 months for AF CA procedures.

### 2.5. Statistical Analysis

A Shapiro-Wilk normality test was performed on continuous variables to examine the normal distribution of each variable. To compare all continuous variables and to test median values between groups, nonparametric pairwise Wilcoxon Rank Sum Tests were performed, with Benjamini-Hochberg correction for multiple testing. For categorical variables, analysis was performed between groups by means of the* χ*^*2*^ test.

Box and Whisker Plots were used to depict the trends in the parameters DAP and FT year by year for all CA procedures.

To assess the impact of experience of the systematic use of NMSs year by year, we reported the median values of the parameters DAP, FT, and PT as histograms, with the main statistically significant differences (*∗*) resulting from nonparametric pairwise Wilcoxon Rank Sum Tests corrected for multiple testing.

Regarding follow-up and long-term effects, Kaplan-Meier analysis of Disease-free Survival and the Log-Rank Test for comparison between groups were used. Statistical analyses were conducted by means of R software, Version 3.4.3 [23]* (The R Foundation for Statistical Computing)*. A P value of P<0.05 was considered significant.

## 3. Results

### 3.1. Total Population

Our study included 1028 pts (620 males, 408 females) (1145 procedures) who underwent CA from January 2007 to December 2016.  Table 1 summarizes the clinical characteristics of this population.

**Figure 1:**
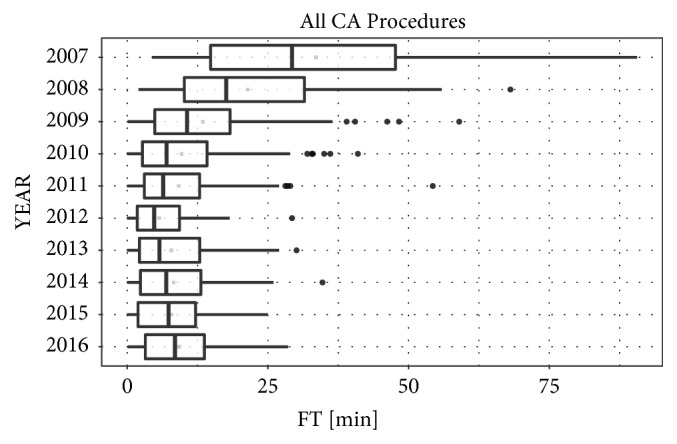
The Box and Whisker Plots show the trend of fluoroscopy time (FT), expressed in minutes [min], in all CA procedures.

**Figure 2:**
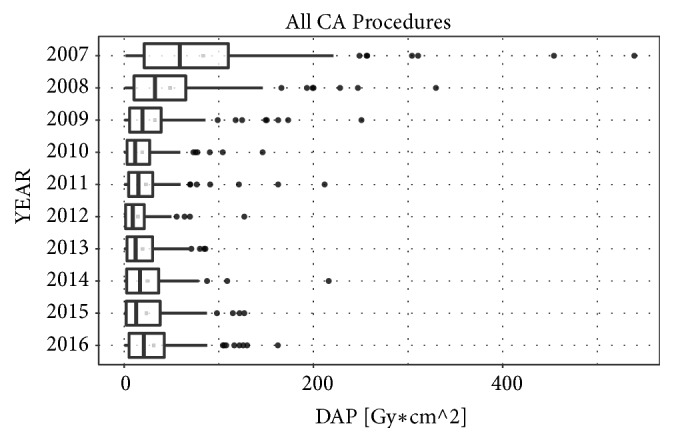
The Box and Whisker Plots show the trend of dose-area product (DAP), expressed in [Gy*∗*cm^2^], in all CA procedures.

Figure 1 shows the change in fluoroscopy time over the years (2007-2016). The median fluoroscopy time of the overall CA procedures fell by 71% from 29.2 min (95% CI [24.2, 37.6]) to 8.4 min (95% CI [7.10, 10.0]) (P<0.001). Over the same period, total X-ray exposure decreased by 65% (Figure 2): from 58.18 Gy*∗*cm^2^ (95% CI [41.8, 71.0]) in 2007 to 20.19 Gy*∗*cm^2^ (95% CI [14.2, 29.7]) in 2016 (P<0.001). This reduction was achieved without significantly prolonging the total procedure time, PT, which displayed a median value of 150 min in 2007 and 176 min in 2016 (P=0.07).

During this period, the maximum reduction in exposure and PT was achieved in the year 2012. The median fluoroscopy time fell by 84% to a value of 4.7 min (95% CI [3.7, 6.0]) (P<0.001), with a concomitant reduction in total X-ray exposure of 85% to a value of 8.48 Gy*∗*cm^2^ (95% CI [5.82, 12.8]) (P<0.001).

This can be explained by the systematic use of NMSs and the increased experience of the operators. After 2012, the entry of new operators with no experience in NMSs led to a slight increase in exposure to fluoroscopy, confirmed by the longer PT.

The percentage of CA procedures performed only with fluoroscopy was 44% in 2007 and 34% in 2008; in the following years, no CA procedures were performed only with fluoroscopy.

### 3.2. Atrial Fibrillation CA Procedures

During the period of our study, 373 AF CA procedures (320 pts) were performed, all of which involved using a single NMS (CARTO). From 2007 to 2016, fluoroscopy time was reduced by 74%, from 49.1 min (95% CI [41.4, 56.6]) to 13 min (95% CI [10.8, 14.6]) (P<0.001) and total X-ray exposure was reduced by 69%, from 138 Gy*∗*cm^2^ (95%CI [73.3, 172.0]) to 43 Gy*∗*cm^2^ (95%CI [32.8, 58.4]) (P<0.001). Over the period considered, different versions of the CARTO mapping system were used for the procedures (CARTO Unix, CARTO XP, CARTO 3); this yielded a further reduction in fluoroscopy, due to the improvement of the software.

During this period, the maximum reduction in exposure and PT was achieved during the year 2012. The median fluoroscopy time fell by 85% to a value of 7.5 min (95% CI [6.0, 9.7]) (P<0.001) with a concomitant reduction in total X-ray exposure of 86%, to a value of 18.9 Gy*∗*cm^2^ (95% CI [14.8, 23.4]) (P<0.001). Again, this can be explained by increased operator experience.

**Figure 3:**
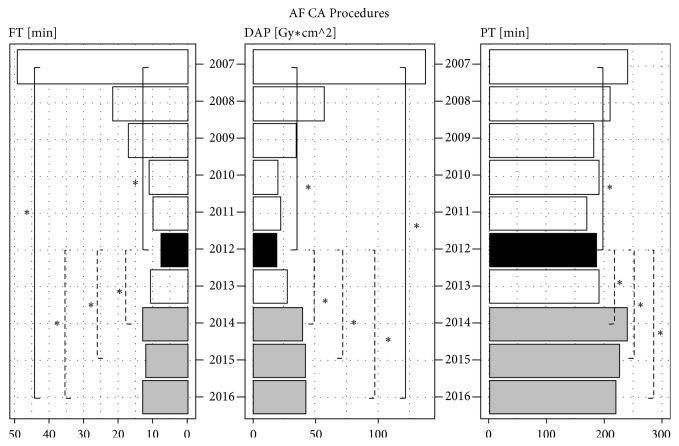
Histograms show the trend of median values of fluoroscopy time (FT), expressed in minutes [min], dose-area product (DAP), expressed in [Gy*∗*cm2], and procedure time (PT), expressed in minutes [min], from 2007 to 2016, with the main statistical significance differences (*∗*) between each year reported as reduction (continuous lines) or as increase (dotted lines), regarding AF CA procedures. In 2012 the maximum optimization of NMSs is reached (median values highlighted with black color), whereas in 2014, 2015, and 2016 a slight increase with respect to 2012 is reported (median values highlighted with gray color).

Figure 3 reports the trends in the median values of DAP, FT, and PT over the years as histograms, with the main statistically significant differences (*∗*). The median values of DAP, FT, and PT declined each year from 2007 to 2012; the histograms for 2012 are highlighted in black. A slight increase in the median values of DAP, FT, and PT in 2014, 2015, and 2016 is highlighted in gray; this was due to the entry of new operators with less experience and the exit of an experienced operator.

### 3.3. Supraventricular Tachycardia CA Procedures

A total of 388 SVT CA procedures (379 pts) were performed during the study period (2007-2016). Like the AF CA procedures, the SVT CA procedures were carried out mainly using a single NMS (EnSite system). During the first period of the study (2007-2011) fluoroscopy time decreased by 93%, from 16.6 min (95% CI [12.2, 21.8]) to 1.2 min (95% CI [0.1, 2.5]) (P<0.001); this was mirrored by a corresponding 92% reduction in DAP, from 12.70 Gy*∗*cm^2^ (95% CI [9.750, 29.60]) to 1.03 Gy*∗*cm^2^ (95% CI [0.106, 2.92]) (P<0.001). This reduction was achieved without prolonging the total procedure time (median value of 80 min in 2007 and 90 min in 2011 P=0.874).

After 2011, the entry of new operators with less experience of NMSs led to a slight increase in exposure to fluoroscopy, the 2016 FT value being 3.0 min (95% CI [1.7, 5.7]) (P<0.001) and the DAP value being 5.94 Gy*∗*cm^2^ (95% CI [2.89, 11.80]) (P=0.005). This is also reflected by the longer PT, with a value in 2016 of 120 min (95% CI [109, 148]) (P=0.002). In 14% of SVT CA procedures, X-ray exposure was zero.

**Figure 4:**
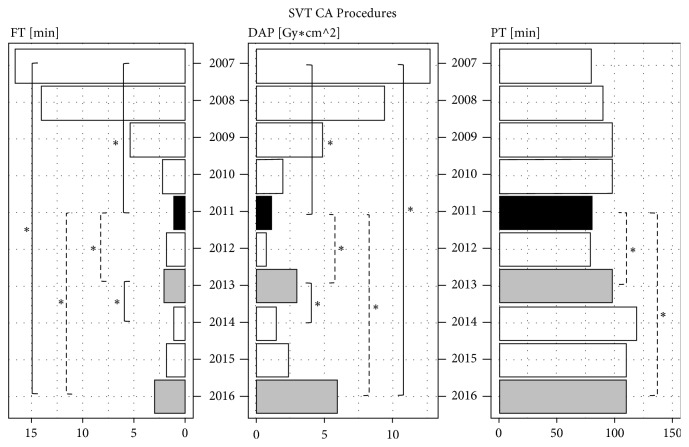
Histograms show the trend of median values of fluoroscopy time (FT), expressed in minutes [min], dose-area product (DAP), expressed in [Gy*∗*cm2], and procedure time (PT), expressed in minutes [min], from 2007 to 2016, with the main statistical significance differences (*∗*) between each year reported as reduction (continuous lines) or as increase (dotted lines), regarding SVT CA procedures. In 2011 the maximum optimization of NMSs is reached (median values highlighted with black color), whereas in 2013 and 2016 a slight increase with respect to 2011 is reported (median values highlighted with gray color).

Figure 4 reports the trend over the years in the median values of DAP, FT, and PT as histograms with the main statistically significant differences (*∗*). The median values of DAP, FT, and PT revealed a reduction in exposure from 2007 to 2011. Regarding SVT CA procedures, 2011 was the year in which the maximum optimization of NMSs was achieved, owing to the experience acquired by the operators. In the histograms, 2011 is highlighted in black. By contrast, the slight increase in DAP, FT, and PT median values from 2013 to 2016, highlighted in gray, can be ascribed to the entry of new, less experienced operators and the exit of an experienced operator.

### 3.4. Procedural Success, Complications and Follow-Up

The postprocedural echocardiogram was normal in almost all patients after CA procedures. However, we had 15 cases of pericarditis and 8 cases of pericardial effusion after ablation (one after transseptal puncture; the procedure was stopped). Furthermore, we had 13 cases of postprocedural femoral hematoma and 2 of venous-arterial fistula. It must be stated, however, that the nature of these complications was not different from the types of complications encountered by our group in the years of work in our EP Lab before the study.

Regarding the procedural follow-up and long-term effects, Kaplan-Meier analysis of disease-free survival was used for both AF CA and SVT CA procedures.

**Figure 5:**
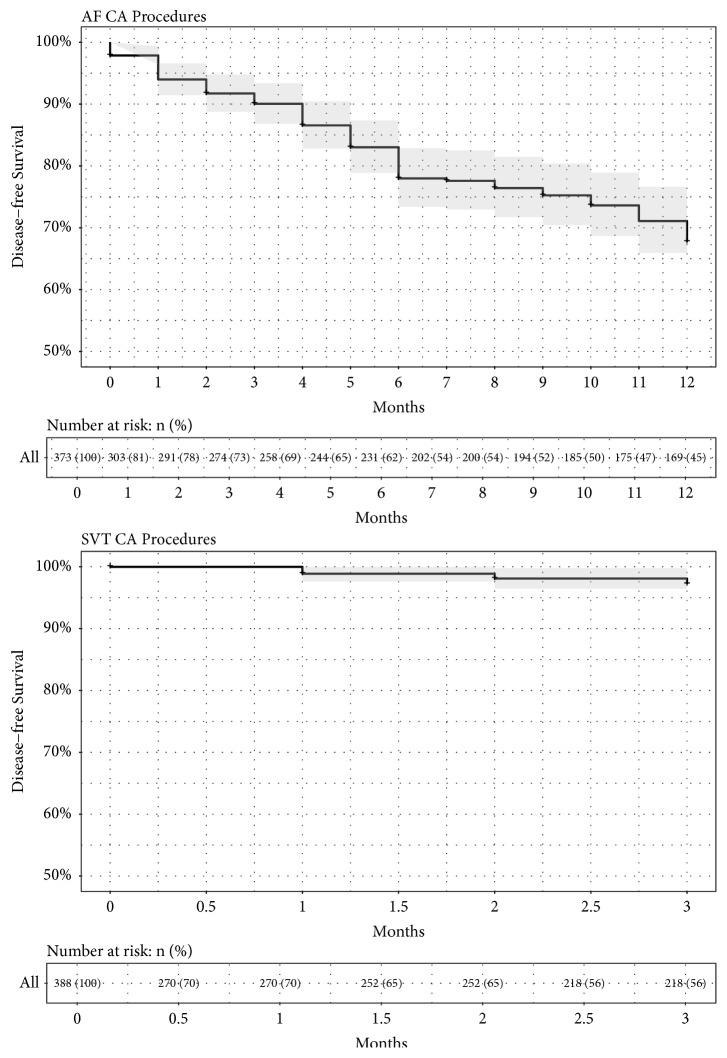
Kaplan-Meier estimates of disease-free survival with 95% confidence interval for all AF CA and SVT CA procedures, with also the table of number of patients at risk reported as number and percentage.

**Figure 6:**
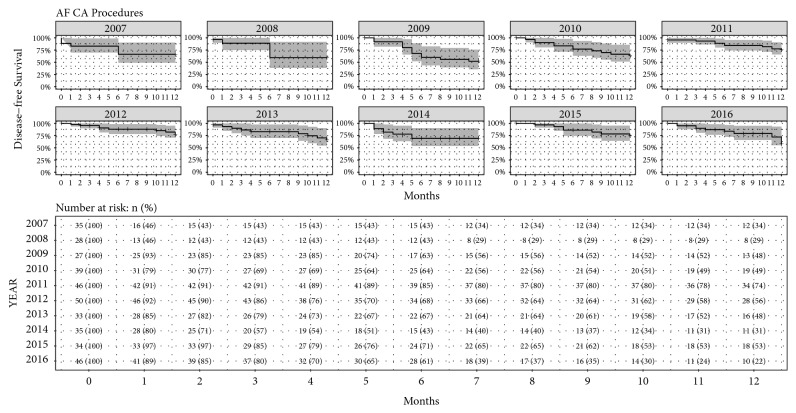
Kaplan-Meier estimates of disease-free survival with 95% confidence interval for AF CA procedures for each year, with also the table of number of patients at risk reported as number and percentage for each year.

**Figure 7:**
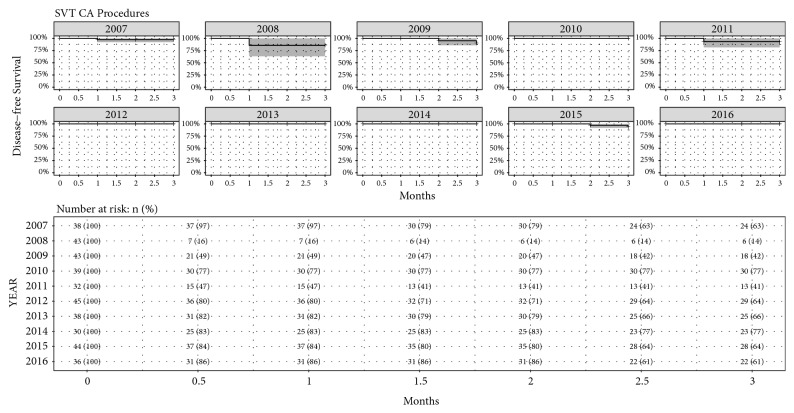
Kaplan-Meier estimates of disease-free survival with 95% confidence interval for SVT CA procedures for each year, with also the table of number of patients at risk reported as number and percentage for each year.

Figure 5 reports the Kaplan-Meier estimates of disease-free survival with 95% confidence intervals for all AF CA and SVT CA procedures, also including the table with the number of patients at risk. At the end of the follow-up period, the estimated probability of disease-free survival was 67.7% (95% CI [62.4%, 73.5%]) at 12 months for all AF CA procedures, and 97.2%(95% CI [95.2%, 99.3%]) at 3 months for SVT CA. For interyear comparison, the Log-Rank Test was used for both AF CA and SVT CA procedures, in order to see if there was a statistically significant difference between the groups.  Figure 6 reports the Kaplan-Meier estimates of disease-free survival with 95% confidence intervals for AF CA procedures for each year, with also the table of number of patients at risk. The Log-Rank Test showed no significance difference between years (P=0.46).  Figure 7 reports the Kaplan-Meier estimates of disease-free survival with 95% confidence intervals for SVT CA procedures for each year, with also the table of number of patients at risk. The Log-Rank Test showed no significant difference between years (P=0.13).

## 4. Discussion

The use of mapping systems was originally limited to the treatment of complex arrhythmias, but it was subsequently shown that their use also for simple arrhythmias may result in simpler and quicker procedures [24–29].

An advantage of the use of a mapping system is the significant reduction in fluoroscopy exposure for both patients and operators [30–34]. In accordance with the ALARA policy (radiation doses “As Low As Reasonably Achievable”) [35] and with the support of our administration, we systematically implemented a mapping system in all CA procedures, starting from October 2008. Present data confirm and extend our previous findings on the feasibility, efficacy, safety, and long-term effect of using an NMS as the main imaging modality to guide the ablation of a wide range of tachyarrhythmias in all types of patients [21].

Our study demonstrated that the extended use of an NMS dramatically reduced radiation exposure in an unselected population of patients, with only slight fluctuations when two trainee operators joined our EP Lab team in 2013 and in 2016.

The initial decrease in radiation exposure was mainly due to the fact that, from January 2007 to October 2008, just under half of the CA procedures were performed by using fluoroscopy as the only imaging modality; subsequently, the systematic use of NMSs was adopted in our EP Lab. Moreover, improvements in NMS software allowed the operator to use less fluoroscopy to check catheter movements, as seen for AF CA procedures. Indeed, after the image integration of the LA, the possibility of visualizing the circular mapping catheter by means of CARTO 3 permitted a further reduction in fluoroscopy in comparison with the use of the CARTO XP Version. Similarly, the implementation of the new software version of EnSite Velocity led to a reduction in fluoroscopy use. In addition, the decrease in radiation exposure can be ascribed to the progressive change of mindset of the electrophysiologist, who is increasingly aware that the use of fluoroscopy during the procedure is sometimes superfluous. In fact, the only use of NMSs does not automatically reduce the radiation dose [36]. Finally, it should be stated that, in the years considered, the working relationship and understanding among the members of our team strengthened, yielding a high degree of confidence and expertise in the use of NMSs, which surely had an influence on the reduction in fluoroscopy.

The data collected in these 10 years of activity at our EP Lab reveal that the systematic use of these new mapping systems is linked to operator learning and experience in a context where these systems are already used. Until 2012, all procedures performed with the aid of NMSs were performed by two operators, who had used these mapping systems from the beginning. Indeed, the maximum optimization of these techniques was reached in 2011 for SVT CA and in 2012 for AF CA. In 2013, an expert operator was replaced by a new operator, who began with the extensive use of NMSs in traditional procedures. At mid-year, the new operator began to take over from more experienced colleagues, even in the most complex procedures, AF CA.

If we consider the lower use of fluoroscopy, as well as a shorter procedure time, to be an indicator of operator experience of NMSs, we can see that the increase in the parameters of DAP, FT, and PT in SVT CA procedures in 2013, in comparison with 2011, reflects the entry of an unskilled operator. It can also be inferred, however, that, within one year, this operator gained experience with traditional procedures, as indicated by the reduced use of fluoroscopy. In 2016, a second new operator, with less experience, started to perform SVT CA procedures, which is again reflected by an increase in the use of fluoroscopy and in procedure time.

After considering the possible factors involved in this significant radiation reduction, an important question remains: why has our EP Lab, despite this 10-year systematic use of NMSs, not succeeded in achieving zero fluoroscopy use? In our opinion, part of the reason for this is that NMNs were created and developed in order to provide a 3D image of the heart for the interpretation of complex arrhythmias. Indeed, the NMS is a dedicated and highly focused mapping tool which does not consider the context of the heart in terms of the organs and structures that surround it (lungs, great vessels, spine, bronchial tree, diaphragm, etc.). By contrast, fluoroscopy provides a less detailed two-dimensional, but bigger, picture. Furthermore, if we subdivide the CA procedure into four parts (venous access, catheter positioning and possible transseptal puncture, electrophysiological study, and ablation, in that order), we can appreciate that the mapping system can significantly reduce radiation exposure in the last two phases, but less in the first two, especially if the operator is less experienced. This is because the mapping system does not allow the operator to visualize wires, long sheaths, transseptal needle, catheter curves, etc. In practical terms, fluoroscopy has its uses throughout the procedure, because of its broader scope.

An extended use of NMSs is associated with increased procedure costs. Nonetheless, this increase is likely compensated by the decrease over the years of radiation induced malignancies. In our previous work on NMSs in children [37] we have shown a certain economic advantage, although the use of NMSs may not be cost-effective in all countries. Additional studies are warranted to assess the cost-effectiveness of an extensive use of NMSs during CA.

## 5. Study Limitations

This study has at least three limitations: first, it was not a randomized multicenter study; second, we did not analyze the cost/benefit effect of this extended use of NMSs and, third, we did not analyze the effect of the reduction in fluoroscopy on the specific risks of exposing patients and medical professionals to radiation.

Moreover, in this study we did not perform an operator-based analysis, describing for each of those involved, independently, trends over the years. This would have allowed identifying a cut-off number of procedures after which performance levels to that of the reference experienced operator.

## 6. Conclusions

The systematic use of an NMS dramatically reduces fluoroscopy time and total X-ray exposure in comparison with the use of fluoroscopy as the main guiding tool in CA procedures. In our experience, this reduction was mainly due to the daily use of NMSs, which enabled EP team members to develop their competence and confidence. We believe that the marked reduction in X-ray exposure reported in our “real-life” study provides a strong argument for the routine use of NMSs in CA procedures and justifies the increased cost. We must also consider that, given the ethical imperative to protect the health of patients and operators, treating cardiac arrhythmias without using an NMS is no longer justifiable. On the other hand, further technological commitment from the industry is needed, in order to overcome the limits of current mapping systems and to achieve total zero fluoroscopy.

## Figures and Tables

**Table 1:** Clinical characteristics of the population of this study.

		TOTAL		2007	2008	2009	2010	2011	2012	2013	2014	2015	2016
		#	%	#	#	#	#	#	#	#	#	#	#
PROCEDURES		1145	100	110	119	102	117	122	125	108	104	114	124

AGE	Mean [years]	55.7	-	53.9	53.3	57.5	54.7	56.5	54.4	55.3	57.1	56.5	58.1
	Standard deviation [years]	16.1	-	16.2	16.8	14.4	16.7	16.22	16.1	16.6	17.3	15.6	14.3

SEX	F	449	60.8	39	55	44	44	45	47	43	39	57	36
	M	696	39.2	71	64	58	73	77	78	65	65	57	88

HEART DISEASE	None	551	48.1	61	61	47	63	58	61	53	42	59	46
	Hypertensive	335	29.3	23	27	28	25	31	44	29	37	36	55
	Valvular	74	6.5	8	15	12	5	10	5	2	6	3	8
	Ischemic	85	7.4	10	6	8	11	7	4	9	14	11	5
	Dilated cardiomyopathy	54	4.7	6	5	4	8	12	3	8	1	1	6
	Congenital	14	1.2	0	5	2	1	0	1	2	1	1	1
	Other	32	2.8	2	0	1	4	4	7	5	3	3	3

LVEF	< 35%	46	4.0	6	1	5	5	5	2	8	5	3	6
	> 55%	917	80.1	89	85	78	97	97	106	77	88	97	103
	35-45%	42	3.7	5	5	4	7	8	2	4	2	3	2
	45-55%	140	12.2	10	28	15	8	12	15	19	9	11	13

ARRHYTHMIA	AF	378	33.0	35	29	27	39	48	50	34	36	34	46
	SVT	435	38.0	49	54	46	46	33	48	39	31	45	44
	AFL/AT	244	21.3	25	32	24	18	27	23	22	27	21	25
	VT	88	7.7	1	4	5	14	14	4	13	10	14	9

F, female; M, male; LVEF, left ventricular ejection fraction; AF, atrial fibrillation; SVT, supraventricular tachycardia; AFL, atrial flutter; AT, atrial tachycardia; VT, ventricular tachycardia

## Data Availability

The data used to support the findings of this study are available from the corresponding author upon request.
